# Characterization of fume particles generated during arc welding with various covered electrodes

**DOI:** 10.1038/s41598-018-35494-1

**Published:** 2018-11-21

**Authors:** K. Yu. Kirichenko, A. I. Agoshkov, V. A. Drozd, A. V. Gridasov, A. S. Kholodov, S. P. Kobylyakov, D. Yu. Kosyanov, A. M. Zakharenko, A. A. Karabtsov, S. R. Shimanskii, A. K. Stratidakis, Ya. O. Mezhuev, A. M. Tsatsakis, K. S. Golokhvast

**Affiliations:** 1 Far Eastern Federal University, Sukhanova Street, 8, Vladivostok, 690950 Russian Federation; 2 Far Eastern Geological Institute, FEB RAS, pr-t 100-let Vladivostoku, 159, Vladivostok, 690022 Russian Federation; 3 Saint-Petersburg State University of Architecture and Civil Engineering, 2-ya Krasnoarmeiskaya Street, 4, Saint-Petersburg, 190005 Russian Federation; 4 Dmitry Mendeleev University of Chemical Technology of Russia, Miusskayasquare, 9, Moscow, 125047 Russian Federation; 5 Laboratory of Toxicology, School of Medicine, University of Crete, Heraklion, 71003 Greece

## Abstract

Arc welding operations are considered to be risky procedures by generating hazardous welding fume for human health. This study focuses on the key characteristics, as well as dispersion models, of welding fumes within a work zone. Commercial and widely used types of electrodes with various types of covering (rutile, basic, acidic and rutile-cellulose) were used in a series of experiments on arc welding operations, under 100 and 150 amps of electric current. According to the results of this study, maximum levels of pollution with particles of PM_10_ fraction occur in the workspace during arc welding operations. Disregarding the types of electrodes used, the 3D models of dispersion of the РМ_10_ particles at the floor plane exhibit corrugated morphologies while also demonstrate high concentrations of the РМ_10_ particles at distances 0–3 m and 4–5 m from the emission source. The morphology of these particles is represented by solid and hollow spheres, ‘nucleus-shell’ structures, perforated spheres, sharp-edged plates, agglomerates of the tree-like (coral) shape. At last the bifractional mechanism of fume particle formation for this type of electrodes is also shown and described. In this article results are reported, which demonstrate the hazards of the arc welding process for human health. The results of the characterization of WFs reported improve our understanding of risks that these operations pose to human health and may strengthen the need for their control and mitigation.

## Introduction

Intense heat typical in welding operations is responsible for high levels of fume concentrations in industrial areas. Fume is comprised of airborne metal or metal oxide particles that have condensed from vapor. In its turn, vapors are generated by the intense high-temperature burning and volatilization of metal, flux and alloying elements^1^. During these processes, 1–3% of electrode mass turns into vapors, and the elemental composition of welding fume (WF) is generally determined by the elemental composition of the electrode and the material being welded^2^. Based on the fact that particle sedimentation of WFs does not occur instantly, differences in this process for nano- and micro-particles explain their prolonged suspension state^3^. Low speed sedimentation of fine particles of WFs (≤0.08 m/s) causes their even dispersion within the workspace, which imposes regulations to ensure the health and safety of such workers^4^. Moreover, these particles are easily affected by the airflow and may spread far beyond the working area^2^, and also absorbed by the welder’s body^5^. The main components of the WFs are oxides of iron, manganese and silicon (~41, 18 and 6%, respectively), as well as chrome^6,7^. Infiltration of toxic compounds of WFs in a human organism through the respiratory tract is linked to dangerous health effects in welders. The biological hazard of WFs due to oxidation of components are well known^5^.

The modernization of safety measures, in turn, is impossible to achieve without detailed information about the formation of the WF (in particular, the fine PM_10_ particle fraction), their morphology and elemental composition, as well as the model of dispersion within the space of the working zone^8,9^. Smaller sizes of WF within the nano-size range (<0.1 µm) exhibit greater risks for human health. Previous studies have demonstrated the ability of nanoparticles to translocate even into the central nervous system (CNS)^10–12^.

According to the literature, the characteristics of WF depend on the type of the electrode covering^2,13–15^. In addition, other studies have demonstrated that the particle sizes and dispersion of WF are dependent on the combination of other parameters, such as welding conditions, methods of welding, as well as methods of analysis^3,7,14–17^. According to the majority of studies, with variable chemical composition of particles, primary particle size of WF range from 10 to 200 nm, and the size of their agglomerates varies from 100 nm to several µm^3,6,7,14,15,17–19^. In addition, it has been reported that the main focus of the study of particle dispersion of WF should be related to the workers’ actual breathing zone^9,20–22^. The possible geometries (3D models) of size distribution of WF have not been reported previously. Today there is no common point of view of the correlation between the welding parameters, such as amperage and levels of WFs; some authors do record reduction of vapor levels when the amperage is increased^13^. According to other sources, the amperage applied to the welding arc is proportional to the temperature of molten metal, which influences the intensity of their vaporization and the formation of vapors (and consequently, fumes)^3^.

This study focuses on the key characteristics, as well as dispersion models, of WFs within a work zone, using the example of arc welding with commercial electrodes with various types of covering. Such studies are essential for an initial health-risks evaluation of electrode toxicity for the purposes of minimization of welding vapors (fumes) levels.

## Materials and Methods

### Sampling methods for the WF within the space of the working zone

All experiments took place in the Welding Department, School of Engineering, Far Eastern Federal University. The experiments were conducted in an isolated room with 60 m^2^ floor space (7.5 m × 8 m), without natural or mechanical ventilation.

**Figure 1:**
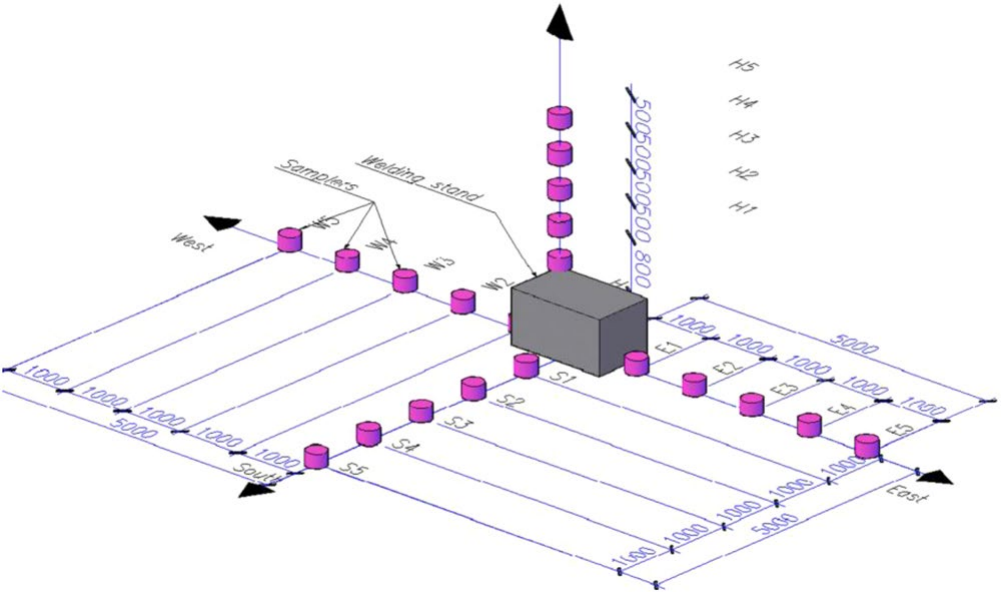
Sampling methods for the WF within the space of the working zone.

Before the initiation of the welding process, plastic (PVC) containers with 2.7 liters of deionized water were placed at the floor line and along the height, as described further down. They were placed at the floor line at the 0.0 mark in three directions (↓S, ← W, → E) and in increments of 1 m from the welding skid (its center is assumed to be the coordinates center (Fig. 1) and along the height ↑H — in increments of 0.5 m from the tabletop of the welding skid (H = 0.8 m from the floor line). During the welding experiments, air samples were collected from 20 different points of the laboratory space (5 for each direction) (Fig. 1). This type of evaluation method has been applied previously^23,24^.

During the experimental procedure, 4 types of popular welding electrodes with different types of covering were used, under amperage of 100 and 150 А (UDSU-251, SELMA, Russia). This experiment was conducted with a three-time repetition using the described electrodes from different manufacturers. Table 1 shows information of the used commercial electrodes, type and thickness of the plate.

**Table 1:** Summary of used commercial electrodes, type and thickness of the plate.

No.	Type and thickness of plate	Type of welding electrode
1	Metal plate VSt-3sp (construction steel), S = 8 mm	MR-3 with rutile covering, Ø3 mm
2		КК-50N Kiswel with rutile covering, Ø3 mm
3		Cho Sun CR-13 with rutile covering, Ø3 mm
4		UONI-13/55 with basic covering, Ø3 mm
5		Bridge Brand J-421 with acidic, Ø3 mm
6	Stainless steel plate, S = 4 mm	EA-395/9-3.0-LD1 E-B20 with rutile covering, Ø3 mm
7		EA-112/15-4.0-LD2 E-B20 with rutile covering, Ø4 mm
8	Metal plate VSt-3sp (construction steel), S = 8 mm	48N-1-LD with basic covering, Ø1 mm
9	Metal plate VSt-3sp (construction steel), S = 8 mm	ESAB OK-46 with rutile-cellulose covering, Ø3 mm
10		MGM-50М with basic covering

The duration of each experiment was determined by the burning time of one electrode (~1 min) and the time needed for complete sedimentation of the WFs (1 h). This time period was selected based on the average time needed for a welder working in one spot during the technological process of production. The laboratory was evacuated until complete sedimentation of particles into the containers (after the burning of electrode). Characterization of the particle-size composition of WF samples, collected in a container with water, implies approximation of the conditions inside the human body in the context of their size and morphology due to possible processes of additional aggregation. This approximation is less applicable when characterizing size distribution of particles directly in the air or after concentrating on filters. Therefore, further results will be presented for the particle size distribution, reflecting the particle size distribution after their absorption by water, i.e. in conditions that approximately simulate their primary contact with the welder’s body.

### Characterization of the WF samples

The histograms of particle-size distribution of WFs after they are deposited on the surface of deionized water were determined by dynamic light scattering (DLS), using Analysette 22 NanoTec plus (Fritsch GmbH, Germany). The measurements of each sample were conducted in Nano (0.01–45.00 µm) and Micro mode (0.08–2000.00 µm) under ultrasound for 30 seconds. Since some of the particles have a difficult geometric form, an ideal match between histograms of particle-size distribution is impossible, with an increase in size range (≥1 µm) these differences will become more significant. Therefore, the histogram was considered correct when the value of Span ([D_90_ − D_10_]/D_50_) differed by <10% from the characteristics of the previous sample (D_10_, D_50_ and D_90_ are the intercepts for 10%, 50% and 90% of the cumulative number, respectively).

The morphology and quantitative chemical analysis of WF were studied on an electron-probe WDS-EDX combined microanalyzer JXA 8100 (JEOL, Japan) equipped with an energy dispersive spectrometer INCA X-Sight (Oxford Instruments, Great Britain).

### 3D-modeling

The 3D-modeling of WFs was carried out based on data of laser nephelometry of particles using specialized AutoCAD software (version J.51.0.0, Autodesk Education Master Suite 2015, Product serial number: 545-89603482). For the development of 3D models, the following algorithm was used:

1. A straight line was plotted from the center of each container, corresponding to the percentage of particles of the size within the range of <10 µm (РM_10_ fraction) in a sample. A straight line was plotted along the ↑Н axis for the containers placed at the floor line, whereas for the containers that were placed along the height the line was plotted parallel to the floor (axis ↓S, ← W, → E).
2. The extreme points of the lines that were plotted from the centers of containers are connected with curved lines. For the containers placed at the floor line - the curved lines intersect the top points of lines that were equally offset from the emission source, the welding skid. For the containers that were placed along the height - the curved lines represent circumferences with radii equal to the length of the straight lines (p. 1).
3. In accordance with obtained data, pp. 1, 2, the planes are plotted as follows: the first one connects the curves for the containers at the floor line and the second one - circles for containers that are placed along the height.

## Results and Discussion

**Figure 2:**
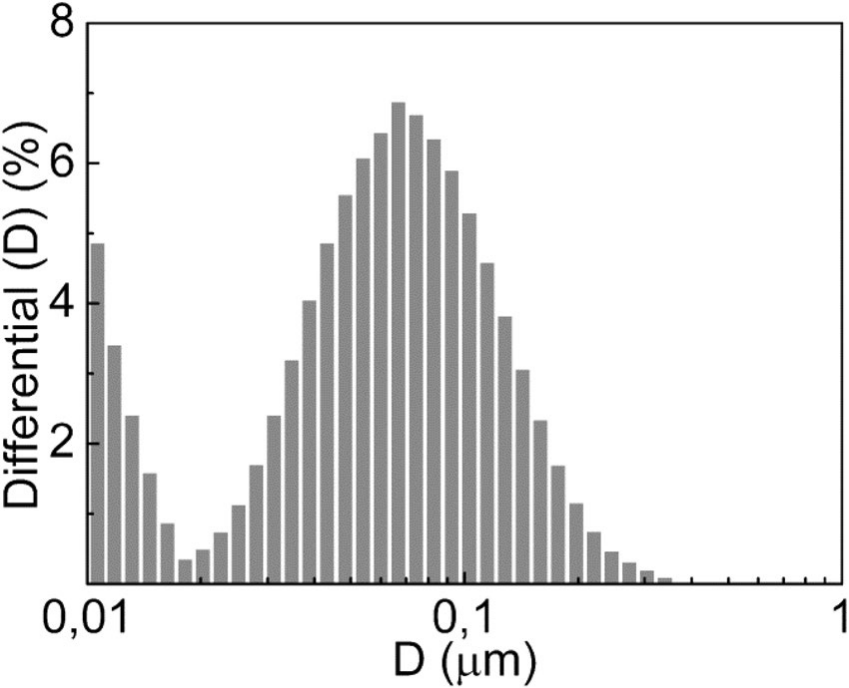
Particle size distribution of WF at ‘Nano’ mode (MR-3 rod with rutile covering).

Based on the results of previously reported studies^14,15,17,23^ that showed typical predominance of micro- and nanoparticles in WFs, the 3D-modeling of clouds was based on the granulometric data, obtained by the ‘Nano’ mode of measurements. It should be noted that depending on the materials that were welded, the median values of particle size distribution (D_50_) varied from 0.06 µm (electrode EA-395/9-3.0-LD1 E-B20) to 94.71 µm (electrode КК-50N Kiswel). This shows that within a radius of 5 m from the source, the particle size after absorption by water varies over a very wide range. In this case, only a fraction of small particles is capable of forming relatively stable aerosols, whereas large particles are susceptible to rapid precipitation if they do not contain cavities. Regardless of the reasons for the formation of large particles (secondary agglomeration in air and water or the formation of sprays), their presence when absorbed by water indicates the possibility of their absorption by the welder’s body. The minimum particle size potentially absorbed by the particle welder’s body at different points of the working zone was determined with the use of the MR-3 electrode with rutile covering (Ø3 mm) (Fig. 2).

**Figure 3:**
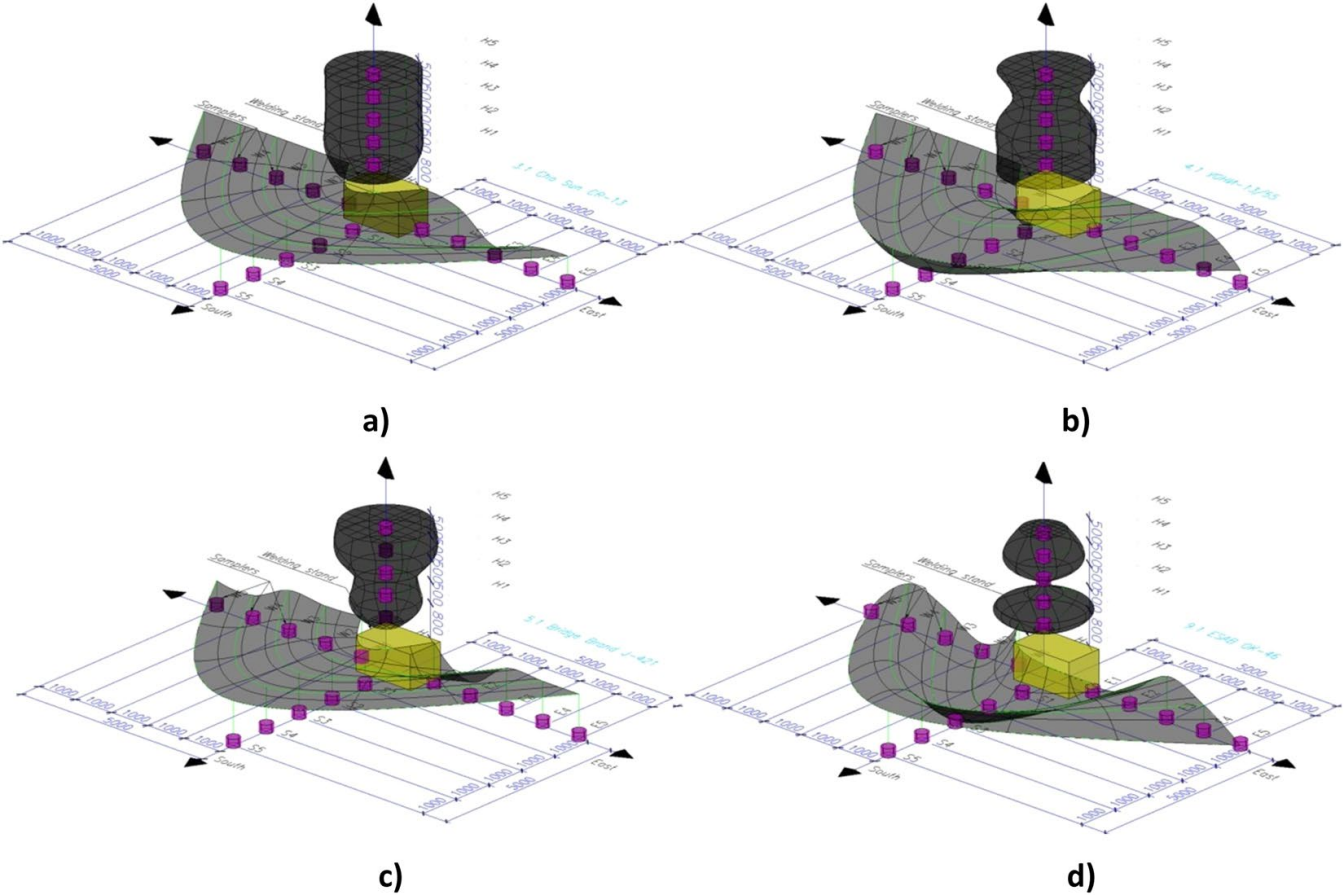
3D models of particle distribution of РМ_10_ fraction of WFs during welding with industrial electrodes Cho Sun CR-13 (**a**), UONI-13/55 (**b**), Bridge Brand J-421 (**c**), ESAB OK-46 (**d**) (metal plates VSt-3sp, S = 8 mm, I = 150 А).

Thus, the peculiarities of formation of fume particles of РМ_10_ fraction within the entire space of the working zone were examined, using commercial electrodes Cho Sun CR-13, UONI-13/5, Bridge Brand J-421, ESAB OK-46 with various types of covering (Fig. 1 and 3, Tables 1 and 2). Table 2 presents the mean values of measurement results. The differences in values do not exceed 12%. According to other reference data, the presence of РМ_10_ particles in the air of workspaces varies within the range 15–80% (depending on the type of the industrial object)^25^. As a conclusion, maximum levels of pollution with particles of PM_10_ fraction occur in the workspace during arc welding operations (Table 2). Figure 3 shows 3D models of РМ_10_ particle distribution of within the workspace when the applied amperage is 150 А and use of various types of covered electrodes. 3D models with applied amperage of 100 А were presented in previous studies^23,24^. These models represent the percentage of particles of РМ_10_ fraction of the total amount of WF at different points of the workspace. Therefore, the addition of percentages of each one of the 3 directions (↓S, ← W, → E) corresponds to the total 100% of WFs. Disregarding the types of electrodes used, the 3D models of РМ_10_ particle distribution at the floor plane exhibited corrugated morphologies. All 3D models demonstrate high concentrations of the РМ_10_ particles at distances 0–3 m and 4–5 m from the emission source (Fig. 3). This peculiarity may be connected with the height of the emission source from the floor line (0.8 m). The fume cloud seems to reach levels of Q (РМ_10_) > 60% even at distances of 5 m from the emission zone when electrodes with rutile, basic and acidic coverings and applied amperage of 150 А are used (Table 1, Fig. 3b). It should be noted, that this entails the pollution of a space of over 280 m^3^ during welding operations, able to be caused just by one electrode (~1 min). Therefore, presence of the supporting working staff within this working zone without protective equipment is dangerous for their health (in accordance with Fig. 1).

**Table 2:** Granulometric characteristics of WF depending on the amperage of arc welding with covered electrodes of various types (metal plates VSt-3sp, S = 8 mm).

	Characteristics	Amperage	S1	S2	S3	S4	S5	E1	E2	E3	E4	E5	W1	W2	W3	W4	W5	H1	H2	H3	H4	H5
ChoSun CR-13	Q(х) (%) P ≤ 10 µm	100 А	13.3	100	37.7	38.2	48.8	10.6	11.0	100	79.1	2.5	35.9	100	31.5	100	100	1.2	100	43.1	0.9	24.1
		150 А	99.9	93.9	100	100	100	99.9	87.8	31.0	59.4	67.4	100	100	100	99.9	100	77.4	100	100	98.4	100
	D_50_ (µm)	100 А	16.4	2.2	12.3	12.6	10.7	14.8	14.9	0.1	3.7	16.2	13.2	0.1	13.3	0.1	0.3	17.3	0.1	12.2	18.0	12.3
		150 А	5.2	7.0	3.6	4.0	4.7	4.2	7.0	13.4	10.8	7.6	0.2	0.1	0.1	3.3	3.1	4.5	0.1	2.2	0.4	0.3
UONI-13/55	Q(х) (%) P ≤ 10 µm	100 А	49.1	32.5	82.3	71.9	73.0	71.3	88.5	100	14.7	36.8	22.3	35.8	18.0	11.9	15.1	55.2	41.5	50.8	72.9	100
		150 А	22.8	100	100	29.6	99.2	99.9	100	100	99.9	99.7	97.0	100	100	99.9	96.0	100	100	100	69.1	100
	D_50_ (µm)	100 А	9.2	12.9	8.2	8.6	8.8	5.2	7.3	4.1	13.3	11.8	14.4	12.7	14.6	15.0	15.0	9.1	11.0	10.2	8.7	2.0
		150 А	13.1	0.2	0.2	14.6	0.2	0.1	2.3	0.3	4.1	0.1	0.7	0.1	3.0	2.2	0.9	0.1	0.1	0.1	8.5	0.4
BridgeBrand J-421	Q(х) (%) P ≤ 10 µm	100 А	99.9	100	67.3	97.3	100	100	100	100	100	1.6	100	54.2	100	99.9	80.9	99.9	97.6	100	22.1	99.8
		150 А	90.8	100	100	100	100	100	41.0	100	100	100	100	94.1	100	60.0	63.9	36.0	75.2	63.9	100	100
	D_50_ (µm)	100 А	2.3	0.5	9.1	3.2	1.0	0.7	0.1	0.1	3.1	19.6	0.1	10.5	1.0	0.1	3.8	5.1	5.7	3.5	13.1	5.1
		150 А	2.2	3.4	2.9	3.8	3.4	3.0	12.1	4.0	0.2	0.8	0.1	5.5	2.8	9.1	8.6	13.1	6.9	10.3	3.2	0.1
ESAB OK-46	Q(х) (%) P ≤ 10 µm	100 А	12.3	100	10.7	99.5	100	89.1	2.4	96.8	100	100	99.9	63.9	100	60.6	99.9	16.0	88.5	100	100	96.4
		150 А	99.6	5.2	12.3	88.0	96.0	23.1	39.2	100	66.5	6.7	100	27.4	75.5	99.7	18.1	21.0	99.6	9.2	78.8	48.7
	D_50_ (µm)	100 А	14.8	2.1	15.2	0.2	2.8	2.2	16.3	0.7	0.1	0.1	3.5	8.2	2.8	10.9	4.5	15.3	6.4	4.6	0.2	6.6
		150 А	0.2	18.9	15.5	2.1	6.8	12.1	12.7	0.1	5.8	15.8	0.1	13.3	5.5	0.4	12.7	16.6	0.1	13.9	4.3	13.6

In Table 3, geometrical types of 3D models (↑H axis) are reported in relevance with the types of covered electrodes and the values of amperage applied^23,24^. It should be noted that the amplitudes of dispersion of WFs at the floor line (↓S, ← W, → E) are proportional to their dispersion geometry along the height (↑H) (Fig. 3).

**Table 3:** Geometrical types of 3D models depending on the type of electrode covering.

Type of electrode covering	Geometry of 3D-profile of WF	
	100 А	150 А
Rutile	Solid of revolution for complex function (jar)	Paraboloid, cylinder
Basic	Hyperboloid (vase)	Hyperboloid
Acidic	Complex system of several domes	Intersecting spheres
Rutile-cellulose	Paraboloid	System of ellipsoids

**Figure 4:**
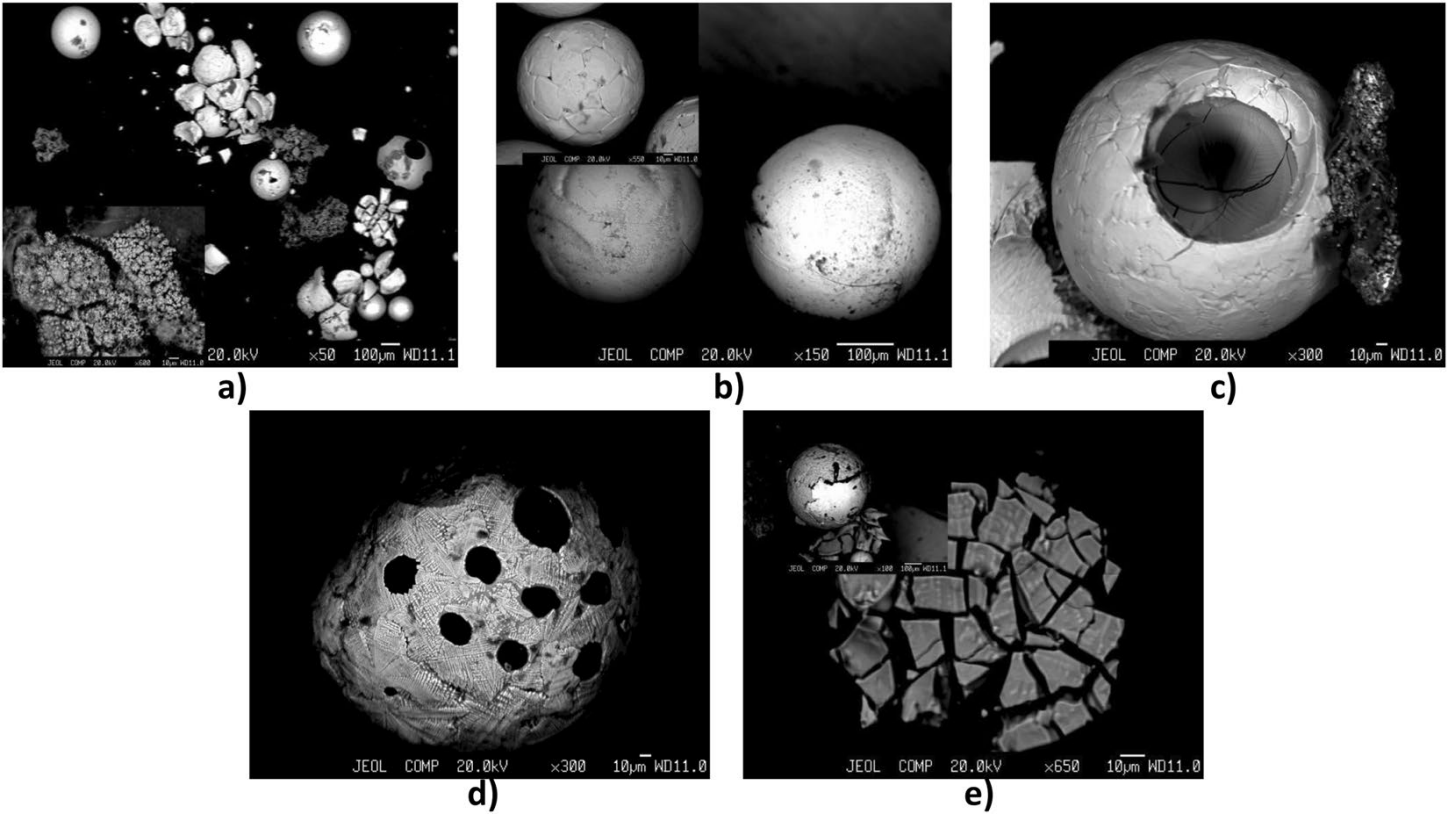
Scanning Electron Microscopy images of the morphological types of solid particulates condensed from vapor during welding using the covered electrode UONI-13/55 of the basic type — general view (**a**), tree-like (coral) (**a**, insert), solid (**b**), hollow (**c**), perforated (**d**), sharp-edged (**e**) and ‘nucleus-shell’ structures (**e**, insert).

**Figure 5:**
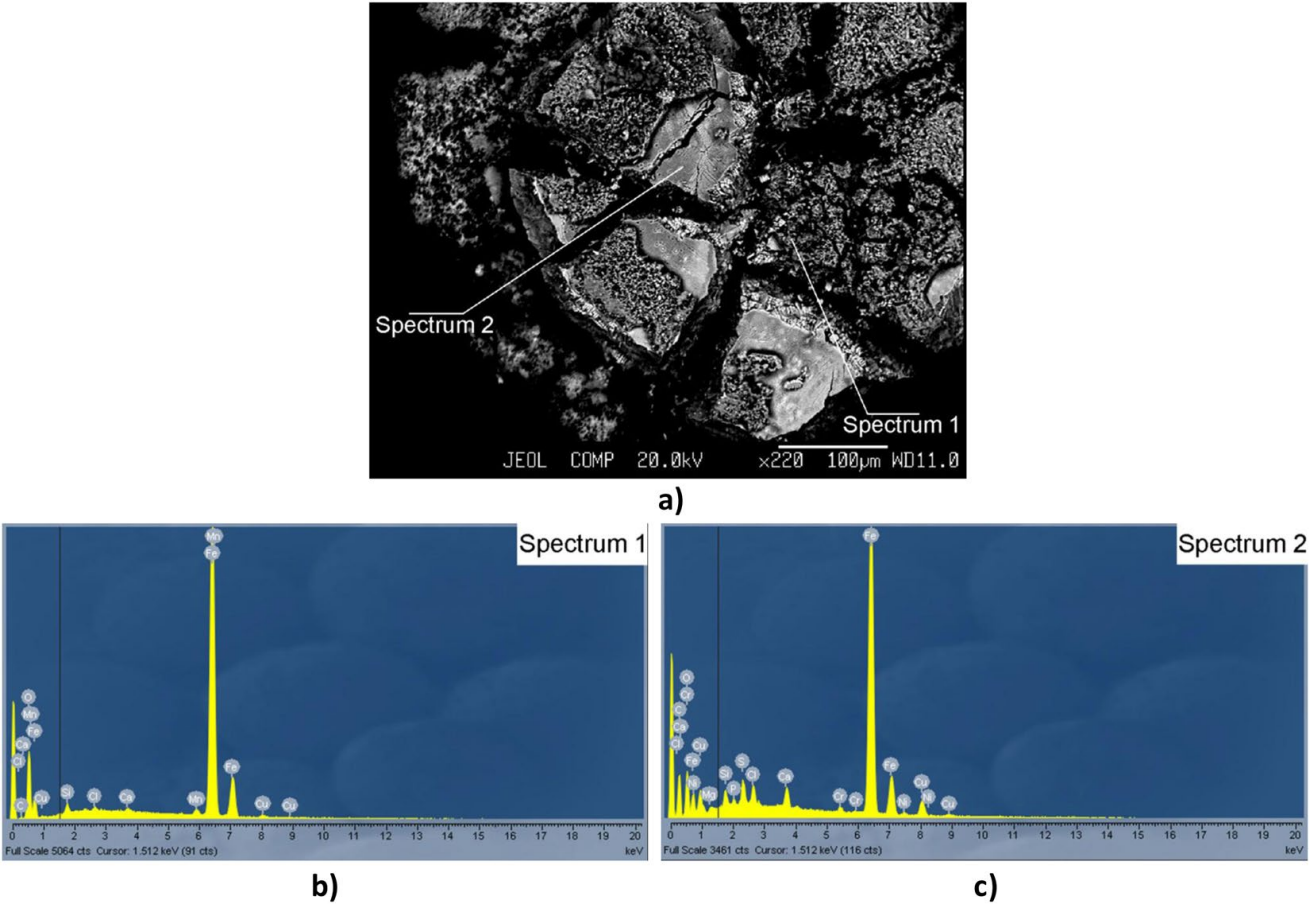
Scanning Electron Microscopy image of WF components (**a**), as well as their element composition – segment spectrum ‘1’ (**b**), and ‘2’ (**c**), accordingly (covered electrode UONI-13/55 of basic type).

In general, when electrodes with rutile and acidic types of covering are used, an increase of amperage from 100 to 150 А causes more even dispersion of the fume cloud in the directions ↓S, ← W, → E. Moreover, use of covered electrodes of acidic type is characterized by minimal difference in values D_50_ and Q (PM_10_) between points of sampling (Fig. 1, Table 2, Fig. 3a,c). In contrast, when electrodes with basic and rutile-cellulose types of coverings are used, the dispersion of particles of the РМ_10_ fraction within the space of the work zone is uneven (Fig. 3b,d)^23,24^. This can be explained by the different intensity of metal vaporization that results from the variableness of the combustible component of the welding vapor that is forming^1,16^. Therefore, an increase in applied amperage causes a decrease in the burning stability of the welding arc. In electrodes with a basic type of covering, the destabilizing factor of the burning arc is the presence of the fluorine ions F^-^ that play the role of arc deionizers^26^. An increase in amperage during the welding process when such type of electrodes are being used, leads to a faster size reduction of particles D_50_, in the area of a worker’s breath (↑H), where this parameter decreases by more than two orders of magnitude (Table 2). Samples collected from different points of the space prove the predominance of nano-sized WF components (<100 nm). This corresponds to previously reported results^1^, showing that the burning of basic type electrodes is less stable in contrast to the rutile ones. The ramp-up of D_50_ with increase of applied amperage from 100 to 150 A is typical for welding using electrodes of rutile-cellulose type. Concerning the electrodes with acidic type covering, no significant changes were observed (Table 2). As a result from the experiments, it is found that the maximum hazard is caused when electrodes with basic covering and high values of amperage applied are used, in contrast when the acidic, rutiles and rutile-cellulose types are used, which do not prove to be that dangerous. Moreover, the biological hazard with basic type of covering, in comparison with non-fluoric electrodes, is increased due to the emission of the toxic gases HF and SiF_4_. The peculiarities of the particle morphology and elemental composition of WF that form during welding with this type of electrodes was also investigated (Figs 4 and 5).

During analysis, the main morphological types of WF were examined and various types of morphologies occurred (solid and hollow spheres, ‘nucleus-shell’ structures^27^, perforated spheres, sharp-edged plates, aggregates of tree-like (coral) shape (Figs 4b–e and 5a). Formation of WF is a process that involves two stages. At first, vaporization of metal in the arc zone takes place leading to the dispersion of the formed vapors with the subsequent competing mechanisms of growth, such as coagulation and condensation^8,9,28^. Thus, the melted microparticles seek minimization of the surfaces free energy, reduction of the contact area up to the spheroidizing moment and reaching then isolation (Fig. 4b–d). In case of nanoparticles, high temperatures lead to irreversible changes in the particle morphology (Fig. 5a). The mass heating of particles and the loss of concrete shape results from the significant activation of the diffusion mass-transfer process. This leads to the formation of agglomerates of tree-like (coral) shape and sizes of up to ~100 μm (Fig. 4a, insert; Fig. 5a)^29^. It should be noted that some microparticles have polycrystalline (ceramic) microstructure (Fig. 4b, insert). The grains of oscillating elemental composition are forming during oxidation of the burning surface of the spherical solid particulates in the atmosphere.

According to data from chemical analysis (Fig. 5b,c), the core of metal composition of the WFs consists of iron Fe, manganese Mn (3rd hazard class), chrome Cr, nickel Ni and copper Cu (2nd hazard class), and calcium Ca, which correlates with the referenced data^6,7,30,31^. The peculiarity of the fume formation during the arc welding process is the combination of the balanced vaporization and unbalanced (combustible) shift of the molten components into fumes. This explains the bifractional formation of WFs (Fig. 5a ‘Spectrum 1’, Fig. 5b). Therefore, the fraction of smaller agglomerates of the tree-like shape is associated with normal vaporization conditions, when the percentage of WF can be represented as a function that depends on the composition of the electrode molten metal and on the values of vapor pressure of its elements^26^. The content of the volatile manganese in this fraction is significant (Fig. 5b). At the same time, the explosive character of the melt evaporation prevents rapid increase in the content of the volatile manganese to the equal partial pressure (Fig. 5c, Scanning Electron Microscopy). Since the manganese compounds are found in great concentrations, it can be concluded that almost all the manganese-containing particles have sizes of the PM_10_ fraction.

Data on the chemical composition and the morphology of the WF is also important for understanding their biological activity and toxicity to human health. The micron solid particulates may damage tissues of internal organs of a human and the particles of the small fraction and their agglomerates of the tree-like (coral) morphology are highly cytotoxic (Figs 4e and 5). The PM_10_ particles (primarily nanoparticles) infiltration of the organism stimulates a protective reaction, which initiates inflammatory processes, including even a development of thrombosis^32^. With reduction of particle sizes, their infiltration abilities increase, as well as the probability of intravasation into the human blood. Ultrafine particle sizes are able to easily infiltrate in lungs through the membranes of the alveolar ridge^10^. The microcirculation abnormalities in human organisms in the end leads to the development of diseases of the cardiovascular system and increases the risks of cancer (leucosis, lung cancer), heart attack and apoplectic attack^33–36^.

Chronic influence of manganese on the human organism can cause genetic mutations and degeneration of the CNS function. This negative effect is similar to Parkinsonism in nature^37,38^. The presence of manganese in covered electrodes of the basic type of the volatile fluorine compounds (KCaF_3_-CaF_2_, Na_2_SiF_6_) and high basicity of the cinder phase promotes an intense flow of alkaline and alki-earthy metal compounds into the WFs (in particular, calcium Ca) (Fig. 5b,c)^5^. The presence in the WFs of the volatile fluorine compounds can lead to the development of asthma^39,40^. Moreover, chrome (Cr) and nickel (Ni) compounds, found in welding wires and welded metals, have been proven to have cancerogenic influence on the human organism (Fig. 5b,c)^41,42^.

Workers of this field need constant biomonitoring of blood and urine for the purposes of evaluation and control of general health risks. Furthermore, warning text and photo messages about the potential risks in the welding zones may help to deliver the information about the hazard levels of ‘industrial sites’ to employees and visitors. In turn, the use of low-fume welding rods and/or elimination of welding fumes by using alternative welding methods, such as friction welding (a solid state process) will make it possible to exclude the negative emissions of welding vapors into the atmosphere.

## Conclusions

The experimental procedures on arc welding, using various commercial covered electrodes, showed some peculiarities of size dispersion of the WF within the workspace. It is shown, that the amplitudes of the wave particles dispersion of the WF at the floor line (↓S, ← W, → E) are proportional to the geometry of their dispersion geometry along the height (↑H). The maximum size reduction of particles with increase in amperage is typical for the welding process using the covered electrodes of the basic type. In the welder’s area of breathing (↑H) the values of D_50_ decrease by more than two orders of magnitude, down to ~0.1 μm. Air samples collected demonstrate the predominance of nano-sized WFs. The morphology of the WF is represented by solid and hollow spheres, ‘nucleus-shell’ structures, perforated spheres, sharp-edged plates, agglomerates of the tree-like (coral) shape.

According to the results of this study, arc-welding operations prove once again to be procedures with high levels of hazard for human health. These results help improve our understanding of risks that these operations pose to human health and may strengthen the need for their control and mitigation. The introduction of 3D modeling of particle size dispersion of WF, during welding arc operations, proves to be an appropriate method for their characterization.

## Data Availability

All data generated or analysed during this study are included in this published article. In addition, further information is available from the corresponding author on reasonable request.
